# Protein Post-translational Modifications in Head and Neck Cancer

**DOI:** 10.3389/fonc.2020.571944

**Published:** 2020-09-30

**Authors:** Hongbo Zhang, Wei Han

**Affiliations:** Department of Oral and Maxillofacial Surgery, Nanjing Stomatological Hospital, Medical School of Nanjing University, Nanjing, China

**Keywords:** post-translational modifications, acetylation, methylation, glycosylation, head and neck neoplasms

## Abstract

Head and neck cancer (HNC) is one of the most common malignant tumors worldwide, and is prone to tumor recurrence and metastasis. At present, surgery combined with radiotherapy and chemotherapy is still the conventional treatment modality for patients with HNC. However, for patients with relapse or metastasis of HNC, the treatment outcome is not ideal, and the prognosis is poor. Thus, it is crucial to deepen the understand of tumor mechanisms. Post-translational modifications (PTMs) refer to covalent binding of small chemical molecular groups to amino-acid side-chain of proteins. Post-translational modification is an important regulator of protein function, and as such, a current research hotspot of epigenetics. In recent years, it has been found that tumor occurrence is often accompanied by the abnormality of PTMs. Indeed, the abnormality play an important role in tumor development, and can be used as a target for tumor diagnosis and treatment. To date, several types of protein PTMs involved in the development of HNC have been reported. This paper reviews the relationship between HNC and several major protein PTMs, including acetylation, methylation, and glycosylation, in order to provide clues for the future application about PTMs in diagnosis and treatment of HNC.

## Introduction

Head and neck cancer (HNC) represents the sixth most common cancer in the world (1). It occurs in the regions of the head and neck, which have complex anatomical structure as well as abundant lymph circulation and blood supply. As HNC involves many organs and is prone to invasion and metastasis, most patients are not at an early stage at their first medical examination. Head and neck cancer can have a great impact on a patient’s appearance and basic physiological functions, such as swallowing, language, and breathing, which could seriously impair their quality of life. At present, there are nearly 700,000 new HNC cases and 380,000 deaths in the world every year (2). In the early stage of HNC, patients without positive lymph node or distant metastases are usually treated by surgical resection combined with local radiotherapy; most of them can be cured. However, the prognosis of patients suffering from advanced HNC is still poor, even if treated with chemotherapy, and shows the five-year survival rate of less than 50% (3). Taken together, it is necessary to actively explore new diagnosis and treatment methods for HNC.

Post-translational modifications (PTMs) are important regulators of protein function. Through covalent binding of small chemical molecular groups to the amino-acid side-chain of proteins, direct changes occur to proteins’ physical and chemical properties, conformation, binding capacity, and function, so as to regulate gene expression at the post-translational level. It is estimated that PTMs can occur in 50–90% of proteins in human body (4). Different PTMs could occur in the same protein, and PTMs of different amino-acid residues on the same protein probably cause different effects. PTMs of various proteins greatly increase flexibility and diversity of functional regulation of proteins to participate in complex life activities. Thanks to the development of mass spectrometry in recent years, we can accurately determine the specific modification of specific proteins, allowing us to understand PTMs and their effects more profoundly. Previous studies have shown that the expression and function of tumor proteins and tumor suppressors are regulated by many kinds of PTMs (5). As HNC is no exception to that, many new targeting possibilities emerge for tumor diagnosis and treatment. Currently, studies on PTMs in HNC are mainly focused on acetylation, methylation, and glycosylation. PTMs are involved in various aspects of HNC, such as proliferation, invasion and metastasis, drug resistance, radio-sensitivity, and anti-apoptosis.

## Acetylation

**FIGURE 1:**
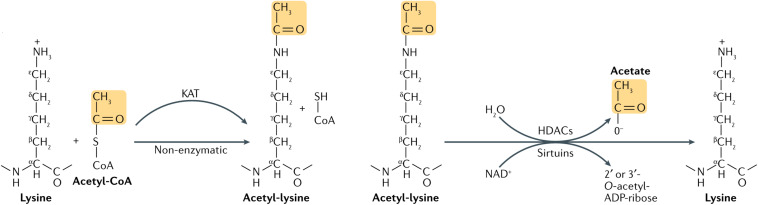
Lysine acetylation occurs through lysine acetyltransferase (KAT)-catalyzed transfer of an acetyl group from acetyl-CoA to the ε-amino side chain of lysine. Alternatively, acetyl-CoA can acetylate lysine non-enzymatically. Acetylation is reversed by Zn2+-dependent histone deacetylases (HDACs), or by the NAD+-dependent sirtuin family of deacetylases. HDAC-catalyzed deacetylation generates deacetylated lysine and acetate, whereas sirtuin-catalyzed deacetylation produces deacetylated lysine, nicotinamide and 2’ or 3’-*O*-acetyl-ADP-ribose (7).

Acetylation was first found on histones in 1964. It was thought to occur mainly on lysine residues of histones, regulated by histone acetyltransferase (HAT) and histone deacetylase (HDAC) (6) (Figure 1). However, advances in proteomics during the past decade have shown that non-histones proteins are also often acetylated, and that acetylation of non-histone proteins is widely involved in key cellular processes related to physiology and diseases, such as gene transcription, DNA damage repair, cell division, signal transduction, protein folding, autophagy, and metabolism (7). During progression of many tumors, the balance between HAT and HDAC is broken, thereby changing the expression level of the acetylation. Through promotion of acetylation in histones and non-histone proteins, histone deacetylase inhibitors (HDACi) can affect cell differentiation, cell cycle arrest, tumor immunity, and apoptosis of damaged cells, exhibiting overall an anti-cancer activity (8). Histone deacetylase inhibitors can be combined with traditional anti-tumor drugs or new targeted drugs to increase the sensitivity of tumor cells to apoptosis and the drug (9). At present, research on acetylation in HNC mainly focuses on HDACi.

Bai et al. (10) found that (S)-HDAC42 had an obvious anti-proliferative effect on oral squamous cell carcinoma (OSCC) cells (Ca922, SAS, HSC-3) and mediated caspase-dependent apoptosis by targeting multiple signaling pathways relevant to cell cycle progression and survival. Specifically, cell experiments *in vitro* showed that (S)-HDAC42 reduced the levels of phosphorylated Akt, cyclin D1, and cyclin dependent kinase 6 and increased the expression of p27 and p21. In addition, (S)-HDAC42 suppressed NF-κB signaling by blocking TNF-α-induced nuclear translocation and activated reactive oxygen species generation. Furthermore, the *in vivo* experiment exhibited a high efficiency in suppressing OSCC tumor growth in Ca922 xenograft nude mouse model. Hehlgans et al. (11) reported NDACI054 could significantly reduce HDAC activity, decrease basal survival, increase acetylation of histone H3, and induce obvious radio-sensitization in tongue squamous cell carcinoma (TSCC) cells (UT-SCC15). A study of two new HDACi’s effect in three chemo-resistant cell lines (HN6, HN12, HN13) of TSCC *in vitro* demonstrated that although two HDACi exhibited low HDAC inhibitory activity, they showed better anti-proliferative activity than cisplatin (>3300 times) at low nanomolar concentrations (12). Moreover, they had higher selectivity and showed no obvious anti-proliferative effects on non-cancer cell lines. Histone deacetylase inhibitors induced accumulation of PTEN protein in a dose-dependent manner, downregulated mTOR to interfere with PI3K/Akt/mTOR pathway, and increased acetylation of histone H3. Jang et al. (13) explored molecular mechanisms of apoptosis in human oral mucoepidermoid carcinoma (MEC) cells (MC3, YD15) induced by sodium butyrate (NaBu), a HDACi with pro-apoptotic activity. They found that NaBu significantly downregulated the expression of the survivin protein, and also reduced the expression of Bcl XL mRNA and protein in human oral MEC cells, leading to caspase-dependent apoptosis. NaBu administration inhibited tumor growth on nude mouse xenograft models bearing MC3 cells at a dose of 500 mg/kg/day, and it did not cause any hepatic or renal toxicity.

However, a phase II clinical trial (14) of Romidepsin, another HDACi, performed in 14 patients with recurrent/metastatic head and neck squamous cell carcinoma (HNSCC) including two primarily localized in oral cavity, six in oropharynx, two in hypopharynx, and four in larynx showed no significant clinical benefit despite high acetylation level of histone H3, reduced Ki67 staining, and decreased expression of HDAC6, 9, and EGFR. Meanwhile, Giudice et al. (15) found that, in addition to reducing the number of tumor stem cells and inhibiting the formation of clonal spheres, HDACi also induced epithelial-mesenchymal transition (EMT) of TSCC cells (HN6, HN13). Another study showed that HDACi induced EMT of nasopharyngeal carcinoma (NPC) cells (CNE2) mainly through increasing the expression of the transcription factor Snail (16), which suggests that HDACi may increase the risk of tumor cell proliferation. Hence, caution is advised in the application of HDACi for tumor therapy, and HDACi may be more suitable for treatment of some non-epithelial tumors.

The findings above suggest that in the treatment of HNC, HDACi may be more useful when combined with other existing therapies. Almeida et al. (17) reported that cisplatin-resistant HNSCC cells (HN6, HN13, Cal27, UM-SCC17B, UM-SCC74A) had active signaling of nuclear factor kappa B (NF-κB), which promoted chemo-resistance of HNSCC by inducing histone deacetylation, reducing BRCA1 level, and thus enhancing genomic instability. Histone acetylation induced by HDACi can prevent NFκB-induced cisplatin resistance and thus enhancing the cytotoxicity following cisplatin treatment. In addition, a newly synthesized HDACi, 13d, can enhance the cytotoxicity of cisplatin to cisplatin-resistant TSCC cell line (Cal27CisR) through caspase-3/7 pathways (18) *in vitro*. Another study demonstrated that radiotherapy combined with HDACi was also feasible in the treatment of HNSCC (19) (SQD9, SCC61, Cal27, SC179).

**FIGURE 2:**
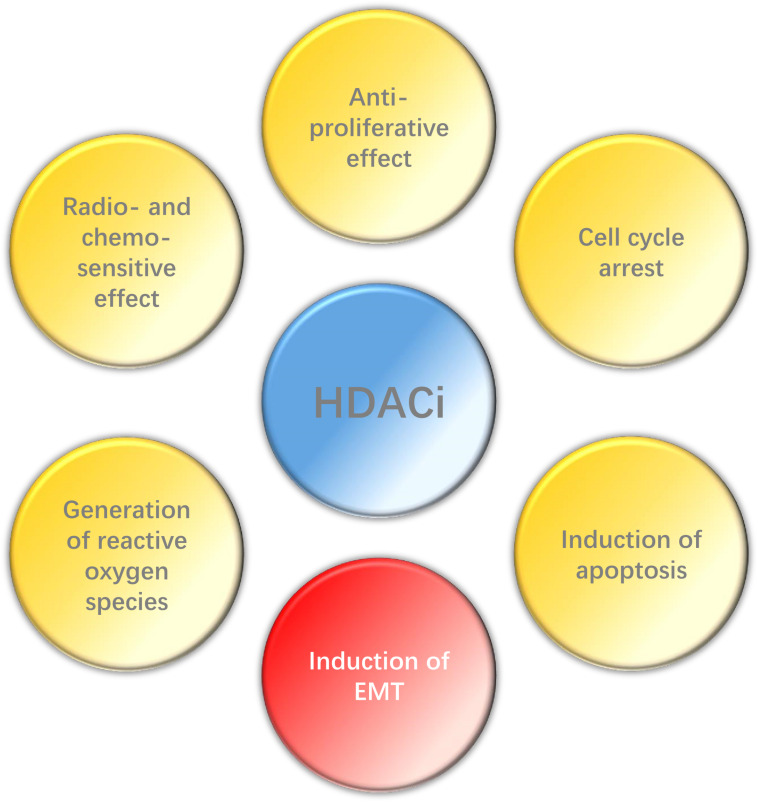
Effect of HDACi on HNC.

In conclusion, HDACi can play a wide range of anti-tumor effects in HNC (Figure 2), such as inhibiting proliferation, promoting apoptosis, enhancing radio- and chemo-sensitization and so on, and also has high selectivity. However, EMT may be induced by HDACi in some epithelial tumors. Histone deacetylase inhibitors have been the first kind of epigenetic-related drug that can be used in cancer treatment. Although their clinical efficacy is still less than optimal, relevant researches still provide valuable epigenetic targets for future anti-cancer strategies. Therefore, it is necessary to carry out further research to explore the potential and possibility of HDACi in the treatment of HNC, espesically when combined with other therapies.

## Methylation

**FIGURE 3:**
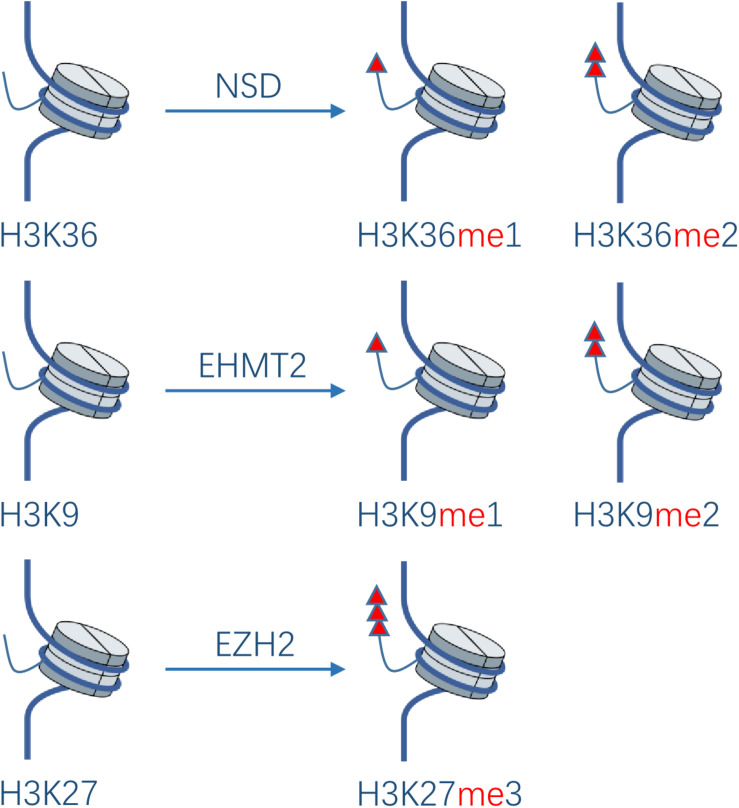
Effect of PKMTs on histone. The NSD family catalyzes the deposition of mono- and di-methyl groups on lysine 36 of histone H3. EHMT2 catalyzes mono- and di-methylation of H3K9. EZH2 catalyzes tri-methylation of H3K27.

Protein methylation usually occurs on arginine or lysine residues. The process is mediated by protein lysine methyltransferases (PKMTs) and protein arginine methyltransferases (PRMTs), which regulate the methylation level jointly with protein demethylases. Although protein methyltransferases (PMTs) regulate epigenetic and transcription mainly through histone methylation, studies have also revealed some non-histone substrates methylated at lysine or arginine residues. It was shown that 95% of the HNSCC patients have genetic or expressional changes of PMTs (20). Based on these observations, PMTs might become a promising new anti-cancer target. In the development of HNC, the main PTMs studied are NSD1, NSD2, NSD3, EHMT2, and EZH2 in PKMTs (Figure 3), as well as PRMT1 and PRMT5 in PRMTs.

The nuclear receptor binding SET domain protein (NSD) family catalyzes the deposition of mono- and di-methyl groups on lysine 36 of histone H3 (H3K36me1, H3K36me2)(20). Loss of H3K36me2 caused by NSD1 inactivating mutations was shown to contribute to HNSCC (Fadu, PCI-4B, SCC4, SKN3) oncogenesis (21). A study showed that 73% of HNSCC sections derived from biopsies of patients with locally advanced disease had moderate or strong overexpression of NSD2 compared with normal epithelial tissues, while the overexpression increased with the development of squamous epithelium to dysplastic epithelium and then to HNSCC (22). NSD3 exhibits similar characteristics (23), which supports their pathogenicity in the initial stage of HNSCC. A high expression of NSD2 is significantly related to the low pathological grade. Knockdown of NSD2 and NSD3 gene via siRNA transfection results in decrease of H3K36me2 and 3, and H3K36me2 levels, respectively, leading to significant reduction of HNSCC cells (UD-SCC2, UM-SCC35, HN-SCC151, PE/CA-PJ15; UD-SCC2, YD-10B, HN13) viability *in vitro* and indicating that they play important roles in H3K36 methylation in HNSCC. NSD3 can also catalyze mono-methylation of EGFR at lysine K721 in the tyrosine kinase domain of EGFR(24). Moderate or strong K721 EGFR mono-methylation was detected in 82% of patients with HNSCC, which may potentiate its interaction with proliferating cell nuclear antigen (PCNA), stabilize PCNA, and promote DNA replication in HNSCC cells. Knockout of NSD3 via siRNA transfection makes HNSCC cells (YD-10B) sensitive to Erlotinib. Namely, it is speculated that lysine K721 methylation may allosterically enhance the affinity of EGFR to ATP and thus decrease the affinity of EGFR to Erlotinib, rendering resistance to the drug. In addition, K721 mono-methylation may also lead to resistance to EGFR inhibitors by potentiating the function of nuclear EGFR, which may be the main resistance mechanism of HNSCC to EGFR inhibitors. Inhibition of NSD3 could improve the therapeutic efficacy of EGFR inhibitors in HNSCC.

Euchromatic histone-lysine N-methyltransferase 2 (EHMT2) can catalyze mono- and di-methylation of H3K9 and inhibit the transcription of the target gene (20). Liu et al. (25) found that knockout of EHMT2 in metastatic HN12 cells via shRNA transfection resulted in decreased expression of EMT markers (N-cadherin and vimentin), restoration of the expression of E-cadherin, and decreased mobility and invasiveness of HN12 cells. Furthermore, the treatment of HN4 cells by BIX01294, an EHMT2 inhibitor, inhibited TGF-β-induced tumor sphere formation and CD44 protein expression. Inhibition of EHMT2 could induce transcriptional upregulation of ERK dephosphatase dual specificity phosphatase4 (DUSP4), leading to inactivation of the ERK pathway and induction of tumor autophagy (26). EHMT2-mediated H3K9 mono-methylation and subsequent transcriptional upregulation of the glutamate-cysteine ligase catalytic subunit promotes glutathione biosynthesis and cisplatin resistance, whereas knockdown of EHMT2 gene via shRNA transfection or treatment with the EHMT2 inhibitor UNC0638 can lead to re-sensitivity of resistant HNSCC cells (SAS-CR, OECM-1) to cisplatin *in vitro* (27). These findings support the idea that EHMT2 may be a reasonable drug target for HNSCC tumor stem cells.

EZH2 is a direct downstream target of tumor-suppressive microRNA miR-26a in NPC, which can catalyze tri-methylation of H3K27. MiR-26a can directly downregulate the translation of EZH2 in NPC, resulting in the decrease of NPC cells (C666-1, HNE-1) proliferation and G1 cell cycle arrest, while the overexpression of EZH2 could rescue the growth-suppressive effect of miR-26a (28). The overexpression of EZH2 via lentiviral transfection in laryngeal cancer cells (AMC-HN8) promotes the cells to enter the S-phase of the cell cycle and promotes their proliferation, tumorigenesis, and resistance to cisplatin (29). EZH2 also plays an important role in invasiveness and EMT of HNSCC. EZH2-mediated H3K27 tri-methylation synergistically represses E-cadherin with HDAC1, 2 and the transcription factor Snail, thus enhancing the metastatic potential of NPC cells (CNE2, HNE-1) (30). Moreover, EZH2 may play a vital role in the maintenance of de-differentiation features in HNSCC cells (SCC25, Detroit 562, Cal27) (31). These results suggest that EZH2, as an oncogene, can promote the cell cycle progression, EMT, chemo-resistance, and de-differentiation of HNSCC, and can be perceived as a reasonable drug target.

Protein arginine methyltransferase 1 can mono- and asymmetrically di-methylate various histone and non-histone substrates. A study on the mechanism of cetuximab resistance suggested that the HNSCC cell lines (OECM-1, FaDu) resistant to cetuximab upregulated the transcription factor Snail and induced direct upregulation of lymphotoxin-β and PRMT1, leading to interaction of lymphotoxin-β and methylated EGFR, and thus activated the resistance to cetuximab (32). Protein arginine methyltransferase 5 can mono- and symmetrically di-methylate both histone and non-histone proteins. It was found that the expression of PRMT5 significantly increased in oropharyngeal squamous cell carcinoma, especially in smokers (33). In the initial stage of HNSCC, the expression of PRMT5 in cytoplasm is upregulated, and the expression level remains unchanged during the whole process of HNSCC. Meanwhile, upregulation of PRMT5 expression in nucleoplasm is related to decrease of E-cadherin and increase of vimentin in tumor specimens, suggesting increased invasiveness of OSCC (34). Furthermore, the expression of PRMT5 in NPC is higher than in paracancerous tissues and is related to poor prognosis. The silencing of PRMT5 promotes radio-sensitivity of NPC cells (5-8F, CNE2), which suggests that the PRMT5 overexpression may be the reason for radio-resistance and poor prognosis in some NPC patients (35).

In general, overexpression of methylation promotes the occurrence and development of HNC, while inhibiting methylation of related proteins can reserve the malignant phenotype of HNC. Therefore, more attention should be paid to the target role of PMTs and their related genes in tumor therapy. So far, there has not been PMTs related drug that can be applied in clinical, and more researches are needed to explore the possibility of its application in HNC treatment.

## Glycosylation

**FIGURE 4:**
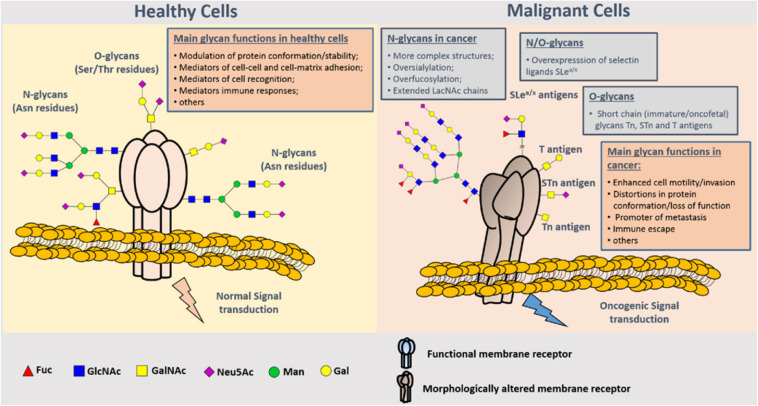
Representation of protein *N*- and *O*-glycosylation in healthy tissues and malignant cells. Protein glycosylation plays a key role in the definition of protein folding and physiological functioning. Glycans contribute to cell–cell and cell–extracellular matrix adhesion, immune cell recognition, among other key biologic processes. Glycosylation is a highly dynamic PTM resulting from the concerted and highly regulated action of several glycosyltransferases in secretory organelles that rapidly changes in response to physiological stimuli. Several *N*- (Asn residues) and *O*- (Ser/Thr residues) glycans may coexist in the same protein backbone, depending on available glycosites and conformational constrains. In comparison to healthy cells, malignant cells tend to present more complex and branched oversialylated and/or fucosylated *N*-glycans. *N*-glycans may also be more extended by LacNAc chains. Conversely, more malignant clones present less complex and immature *O*-glycans, namely the Tn, sTn, and T antigens that may be also found in oncofetal tissues. Some cancer cells may also express selectin ligands sLea/x as terminal structures of both *N*- and *O*-glycans. These structural alterations at the cell-surface favor more motile and plastic cell phenotypes, invasion, lymphatic and hematogenous dissemination, and immune evasion. By impairing normal functions of cell-surface receptors, cancer-associated glycans also interfere with normal intracellular signaling transduction pathways toward the activation of oncogenic features (37).

Glycosylation of proteins is the process of glycosyltransferase-mediated transfer of glycosyl to protein and formation of glycosidic bond with amino-acid residues on the protein, which mainly occurs in endoplasmic reticulum and Golgi body. Glycosyltransferase and glycosidase play key roles in glycosylation. Glycosylation of proteins in mammals can be divided into two types: *N*-glycosylation and *O*-glycosylation. *N*-glycosylation involves formation of a covalent bond between *N*-sugar chain and free NH2 group of the aspartic acid of the protein, whereas *O*-glycosylation encompasses covalent bonding between the *O*-sugar chain and the free OH- group of serine or threonine of the protein. It has been shown that the change of glycosylation influences the progression and metastasis of tumors, and that abnormal glycosylation affects cell signal transduction, adhesion, and migration (36) (Figure 4). Glycosyl and glycoconjugated proteins regulate immune response in cancer cells. Glycan-based biomarkers are effective tools for clinical diagnosis and treatment of cancer. Moreover, glycosylation of proteins plays an important role in chemo-resistance (37), which was confirmed in the study that used tunicamycin to inhibit glycosylation and thereby enhanced the effectiveness of chemotherapy (38).

Glycosylation may play an anti-apoptosis role in HNC. A study (39) showed that cellular prion protein (PrP^C^) *N*-glycosylation could enhance the anti-apoptosis ability of OSCC cell line (HSC-2), while the anti-apoptosis ability was abolished under the tunicamycin-induced *N*-glycosylation inhibition. It indicated that inhibiting overexpressed N-glycosylation of PrPC in HNC cells may be a potential therapeutic strategy. Glycosylation may promote HNC invasion. DPAGT1 gene encodes dolichol-*P*-dependent *N*-acetylglucoseamine-1-phosphate transferase (DPAGT), a key regulator of the metabolic pathway of protein N-glycosylation. It has been found that DPAGT1 is overexpressed due to the abnormal activation of Wnt signal in oral cancer cell lines (Cal27, A253) (40). Another study (41) demonstrated that *N*-acetylgalactosaminyltransferase-2 (GALNT2) is often highly expressed in OSCC, especially in cancer cells at the front of invasion. GALNT2 overexpression enhances the invasive potential of OSCC cells (SAS) via modifying O-glycosylation and activity of EGFR, suggesting that targeting GALNT2 could be a promising approach for OSCC therapy. Glycosylation is also expected to be used in the early diagnosis of HNC. Inspired by the aberrant glycan expression in oral cancer tissues, a study (42) applied legume protein wheat germ agglutinin (WGA) to selectively recognize sialic acid and *N*-acetylglucosamine residues, and detected atypical glycosylation via imaging of fluorophore-conjugated lectins. The results demonstrated that WGA fluorophore probes can successfully yielding statistically higher fluorescence in OSCC tissues, which exhibited its promising ability to distinguish cancerous tissues from pathologically normal tissues. This finding provided a new avenue for early diagnosis and tumor boundary determination of OSCC. Glycosylation is also related to radiation resistance. For example, Dong et al. (43) reported that radio-resistant laryngeal carcinoma cell line (Hep-2max) presented higher core 1-type *O*-glycans than the sensitive one (Hep-2min) and inhibition of *O*-glycan biosynthesis can increase radio-sensitivity. Core 1 β1,3-galactosyltransferase (C1GALT1) modifies O-glycan on integrin β1 and regulates its activity. Overexpression of C1GALT1 via siRNA transfection in Hep-2min cells enhances cell mobility, invasiveness, and radiation resistance, whereas knockout of C1GALT1 in Hep-2max cells inhibits these malignant phenotypes. In this context, O-glycosylation is related to inherent radio-resistance of laryngeal cancer and can regulate migration and invasion of laryngeal cancer cells. These findings suggest that C1GALT1 may be a potential target for the treatment of laryngeal cancer.

## Conclusion

Various lines of evidence have proven that protein PTMs play an important role in the development of HNC, and that they are involved in the regulation of HNC proliferation, invasion and metastasis, chemo-resistance, radio-sensitivity, anti-apoptosis and so on. According to their characteristics, different PTMs can be selectively inhibited or promoted to play anti-cancer effects. With advances in research, many regulatory factors of PTMs with target significance have been found, which have shown preliminary value for clinical treatment. Compared with chemotherapy and radiotherapy, PTMs-related drugs have the advantage of good targeting and high safety, but their therapeutic effects need verification in further clinical research. It is plausible that the combination therapy of PTM regulators and other existing therapies may present a feasible direction for their future clinical application. Except several major PTMs, the role of other PTMs in the development of HNC is poorly understood. With the discovery of an increasing number of PTMs and their specific sites (44), more research needs to be carried out to explore their roles in HNC and unravel molecular mechanisms, so that new clues beneficial to the diagnosis and treatment of HNC can be obtained.

## Author Contributions

HZ contributed to reviewing and classifying the literature, and wrote the first draft of the manuscript. WH contributed to guidance and general oversight. Both authors contributed to the manuscript revision, read, and approved the submitted version.

## Conflict of Interest

The authors declare that the research was conducted in the absence of any commercial or financial relationships that could be construed as a potential conflict of interest.
